# Food insecurity in women with mental illnesses attending a psychiatric hospital in KwaZulu-Natal

**DOI:** 10.4102/sajpsychiatry.v31i0.2342

**Published:** 2025-05-16

**Authors:** Precious S. Dimba, Shamima Saloojee, Vuyokazi Ntlantsana, Sibongile Mashaphu

**Affiliations:** 1 Department of Psychiatry, School of Clinical Medicine, Faculty of Health Sciences, University of KwaZulu-Natal, Durban, South Africa

**Keywords:** food insecurity, quality of life, women with mental illness, psychiatric hospital, KwaZulu-Natal

## Abstract

**Background:**

Food insecurity is a problem for many people globally. Women and people living with mental illnesses are at a particular risk. There is limited information regarding food insecurity in women living with mental illnesses from South Africa.

**Aim:**

To describe the prevalence of food insecurity, its association with socio-demographic and clinical factors, as well as quality of life (QoL) in women with mental illnesses attending a psychiatric hospital.

**Setting:**

King Dinuzulu Hospital Complex in eThekwini KwaZulu-Natal over a 6-month period.

**Methods:**

A researcher-designed questionnaire was used to collect socio-demographic and clinical data, while the Household Food Insecurity Access Scale (HFIAS) and the World Health Organization QoL questionnaire (WHO QOL-BREF) were used to collect data on food insecurity and QoL, respectively.

**Results:**

The 123 participants had a mean age of 50 years (standard deviation [s.d.] ± 13.89), and an overall prevalence of food insecurity of 47.1%. In a bivariate analysis, food insecurity was significantly associated with younger age (*p* = 0.02), having no monthly household income (*p* = 0.01), a comorbid psychiatric diagnosis (*p* = 0.02) and a poorer overall QoL (*p* < 0.001).

**Conclusion:**

Women with mental illnesses had a higher prevalence of food insecurity, with an associated poorer QoL. Additional measures are required to improve food security in this vulnerable group.

**Contribution:**

This study found that women with mental illness had more than twice the prevalence of food insecurity than the general population in KwaZulu-Natal.

## Introduction

Food insecurity is a global problem, but remains a particular challenge for many developing countries, which are faced with widespread inequality and poverty.^1,2,3^ Owing to the coronavirus disease 2019 (COVID-19) pandemic restriction measures, there was a sharp increase in food insecurity in 2020 compared to 2019.^4^ However, even pre-pandemic, over 676 million people in Africa, or approximately half (52.5%) of the population, experienced moderate to severe food insecurity,^2^ with an estimated one in four people who do not have enough food living in sub-Saharan Africa (SSA).^5^ The contention that colonisation in Africa fundamentally disrupted existing food security systems^6^ coupled with natural disasters, wars and climate change, have resulted in a lack of available, accessible and affordable food for many people.^7^

Food security is defined as a situation where people have access to enough food for an active, healthy lifestyle.^8^ Another definition is the assured ability to acquire nutritionally adequate and safe food that meets cultural needs and is acquired in a socially acceptable way.^9^ Levels of household food insecurity range from 26% to 29% among the general population in South Africa (SA),^10,11^ with a study reporting that 47% – 53% of black South Africans are affected by poverty, the largest population of poor households being in KwaZulu-Natal (KZN) province.^12^

Limited access to food is hypothesised to be the result of household poverty, rather than an absolute shortage of food.^13^ Studies found that food insecurity was associated with household income,^14,15^ employment,^7,16^ gender,^17,18^ single parent households,^19,20^ quality of life (QoL)^21^ and the accessibility of material resources within households.^13,22,23^ Certain sub-populations, such as women^18,24,25^ and the mentally ill, are particularly vulnerable to food insecurity.^26,27,28^

In SA, women are at particular risk of food insecurity because of their higher levels of unemployment, lower levels of tertiary education^29^ and greater child-care and household responsibilities, which impact on their ability to work and earn a living.^30^ Female headed households in SA, specifically those of black women, were reported to be poorer and more household insecure than those headed by males.^31,32,33^

Persons living with mental illnesses are particularly vulnerable to food insecurity,^26,34,35^ with evidence of a bidirectional relationship between the two that is, having a mental illness may be a precursor for food insecurity,^26,35^ while being food insecure may increase the risk of developing a mental illness.^11,36^ A meta-analysis comprised of 16 studies from predominantly high-income countries reported that the prevalence of food insecurity in persons with severe mental illnesses (SMI) was 41% compared to 17% for those without mental illnesses from the corresponding general population.^34^ In addition, adults with SMI were 3.31 times more likely to experience food insecurity than those without.^34^ Results from the South African Stress and Health (SASH) study showed a significant association between food insufficiency and having a 12 month and lifetime diagnosis of Diagnostic and Statistical Manual of Mental Disorders, Fourth Edition (DSM IV) anxiety disorder.^11^

Possible explanations for the higher prevalence of food insecurity in individuals with mental illnesses include the high rampancy of unemployment,^35,37^ disparity in wages for the same level of employment^18^ and the disproportionate pervasiveness of mental illness in women.^35^ In terms of gender disparities, women are disproportionately affected by common mental disorders (CMDs) as well as co-morbid mental disorders.^38,39^

Research from high income countries confirmed the high prevalence of food insecurity in women with mental illnesses. A scoping review of 39 studies reported an association between food insecurity and poorer mental health,^24^ and a study conducted across 75 hospitals in the United States (US), found that mothers who were depressed had 70% higher odds of energy insecurity than those who were not.^40^ Of concern is that maternal depression was reported to be an independent risk factor for household food insecurity.^41^

Limited literature was found in low- and middle-income (LMIC) countries, with only two studies investigating food insecurity and mental illness in women. In SA, a study from Cape Town found that there was a statistically significant association between food insecurity, depression and suicidality in the postpartum period.^42^ A study in Afghanistan found that food insecurity was associated with common mental health problems among a sample of reproductive-aged women.^43^ The relationship between food insecurity and mental illness in women is, however, complex, because of an array of other issues, such as the experience of gender-based violence,^24^ unstable housing^40^ and life transitions, including pregnancy.

Quality of life is regarded as the richness of the individual’s personal experience, including their social, occupational and interpersonal functioning,^44^ the construct including the individual’s subjective satisfaction with their life.^44^ An inverse relationship has been reported between food insecurity and QoL,^11,45^ with both being associated with increased healthcare utilisation and mortality among individuals with mental illnesses.^24,46^

There is limited information regarding food insecurity in women living with mental illnesses generally,^47,48,49^ with a dearth of information from Africa. The aim of this study was therefore to determine the prevalence of food insecurity, and its association with socio-demographic factors, clinical factors and QoL in women with mental illnesses attending a psychiatric hospital in KZN province.

## Research methods and design

### Study design and setting

This quantitative, cross-sectional survey was conducted at King Dinuzulu Hospital Complex (KDHC), a public hospital, previously known as King George V Hospital, situated in Springfield, a low- to middle-income area of the eThekwini Municipality. In addition to general health services, the hospital offers specialised services in psychiatry, orthopaedic spinal surgery and multi-drug resistant tuberculosis. This study was conducted at the psychiatry unit, which provides both in-and out-patient psychiatric services.

### Study population

The study population consisted of women with mental illnesses who were attending KDHC from within the eThekwini Municipality and further afield. Patients are usually referred from primary healthcare facilities and the adjacent district hospital for assessment and treatment, and are provided with care through the out-patient clinic by appointment, or by being admitted to the psychiatric ward, where their treatment commences. Once stable, they are discharged, with out-patient appointments needing to be kept monthly to collect their medication or to see a psychiatrist. The services are free, with most patients being poor and unable to afford private healthcare. This is also reflected in their living circumstances, as many are unemployed, which can affect their ability to have adequate food for themselves and their families. The study population is likely to be mainly Indian because of the area of the hospital, which serves mainly the Indian and coloured community.

### Data collection instruments

Three data collection tools were used in this study, these being a researcher designed socio-demographic and clinical data questionnaire, the Household Food Insecurity Access Scale (HFIAS) and the World Health Organization QoL questionnaire (WHO QOL-BREF). Demographic and clinical data were collected by the principal investigator (PI) using a structured questionnaire designed for the study. The socio-demographic questionnaire collected data on age, level of education, employment, marital status, number of dependents, residential area, and grant recipient status. The clinical data collected related to clinical diagnosis, duration of illness, comorbid medical or psychiatric conditions.

The main outcome variable was food insecurity, which was measured using the HFIAS, which was developed by the USAID Food and Nutrition Technical Assistance (FANTA) project.^50^ It is a nine-item questionnaire that has been utilised and validated in a National South African food insecurity survey from KZN.^51^ The scale assesses three domains of food security,^51^ namely, anxiety and uncertainty about household food supply, insufficient food quality and insufficient food intake, and physical consequences. Food insecurity is divided into four categories: secure, mildly, moderately and severely insecure, with the insecure groups being combined into one, the food insecure group.

The WHOQOL-BREF is a 26-item questionnaire that explores four domains related to QoL, that is physical health, psychological, social relationships and environment, and is a valid and reliable cross culturally sound tool.^52^ Both the WHOQOL-BREF and WHOQOL-100 (from which the WHOQOL-BREF was adapted) were developed using cross-cultural, multinational studies, across 15 countries and 30 centres globally.^52^ Responses to the questions are scored using a 5 point-Likert scale, ranging from very poor to very good, or very dissatisfied to very satisfied, as appropriate.

### Data collection process

Data were collected over a period of 6 months, from June 2023 to December 2023, by which time the pandemic was over and things had returned to normal. Doctors working at the KDHC psychiatric unit were briefed regarding the study protocol to identify women for possible inclusion. Women with mental illnesses who met the inclusion criteria were referred by these doctors to the PI, who was responsible for informing them about the study and obtaining their consent to participate. Participants were interviewed by the PI who asked them to read the questions on the three data collection tools that had been uploaded onto an electronic device, their answers being recorded onto the device with a data collection tool called REDCap. The PI is bilingual in English and IsiZulu, and was at hand to help those participants who required such assistance with the use of the device and clarity with the questions, which was provided in English.

### Sampling

Convenience sampling was used to identify women for inclusion, with an estimated proportion of 0.3% of the women who attended the clinic during the year being required, a desired precision of 8% and a 95% confidence interval (CI), which resulted in a sample size of 123 participants being regarded as adequate.^53^

Participants needed to be 18 years and older, able to read and write in English or IsiZulu, stable outpatients or inpatients, who were already stable and discharged in accordance with the *Mental Health Care Act* of 2002^54^ that is, in possession of a Form 3, waiting to be fetched from the hospital by their families, and able to give consent to participate. Involuntary outpatients and those that could not provide consent were excluded from the study. Of the 124 women who were approached, 123 consented and were enrolled in the study, this being regarded as adequate.

### Data analysis

Statistical analyses were conducted using Stata 18, with descriptive analyses, frequencies, means and medians being used to describe categorical and continuous data for all data sets. Bivariate analysis was conducted to test for association between the food secure and insecure groups, while the two-sample *t*-test was used for association with continuous variables and the Wilcoxon rank-sum test was applied for non-parametric or non-normally distributed variables. The Pearson’s Chi-squared test was used for categorical variables with the Fisher’s exact test being used when the numbers were less than five.

### Ethical considerations

Ethical clearance to conduct this study was obtained from the University of KwaZulu-Natal Biomedical Research Ethics Committee (No. BREC/00005109/2022). Permission to conduct the study at KDHC, which is a government facility, was obtained from the KZN Provincial Department of Health, with approval also obtained from the hospital manager. This study was conducted in accordance with the South African Good Clinical Practice Guidelines, namely the ethical principles, risk, burdens and benefit, right and safety of participants, informed consent, ethics review, appropriate management of study data, and exclusion of minors. Written informed consent was obtained from all participants in English or IsiZulu.

## Results

A total of 124 women were approached to participate in the study, only one participant having declined, with 123 female participants being included in the study. The mean age of participants was 50.49 years with standard deviation (s.d.) ±13.76 (Table 1), with most being of Indian ethnicity (48.78%), single (54.47%) and living with their parents or siblings (43.9%). The majority were unemployed (83.74%), and while most relied on social (26.83%) or disability grants (43.09%) for their monthly household income, 15.45% had no source of monthly income.

**TABLE 1:** Socio-demographic variables (*N* = 123).

Variable	*n*	%	Mean	s.d.	Median	IQR
**Age (years)**	-	-	50.49	13.76	-	-
**Race**						
Black or African people	32	26.02	-	-	-	-
White people	7	5.69	-	-	-	-
Indian people	60	48.78	-	-	-	-
Coloured people	24	19.51	-	-	-	-
**Marital status**						
Single	67	54.47	-	-	-	-
Married	32	26.02	-	-	-	-
Divorced	24	19.51	-	-	-	-
**Living with**						
Alone	9	7.32	-	-	-	-
Spouse and/or children	27	21.95	-	-	-	-
Parents or siblings	54	43.90	-	-	-	-
Other†	33	26.83	-	-	-	-
**Area of residence**						
Urban	115	93.50	-	-	-	-
Rural	8	6.50	-	-	-	-
**Primary diagnosis**						
Schizophrenia spectrum	65	52.85	-	-	-	-
Major Depressive Disorder	32	26.02	-	-	-	-
Bipolar Mood Disorder	12	9.76	-	-	-	-
Anxiety Disorder	3	2.44	-	-	-	-
Other	11	8.94	-	-	-	-
**Duration of illness**	-	-	-	-	12.00	6.00, 19.00
**Comorbid diagnosis psych only**						
No	108	87.80	-	-	-	-
Yes	15	12.20	-	-	-	-
**Comorbid medical diagnosis**						
No	117	95.12	-	-	-	-
Yes	6	4.88	-	-	-	-
**Highest grade passed**	-	-	-	-	12.00	10.00, 12.00
**Tertiary education**						
No	90	73.17	-	-	-	-
Yes	33	26.83	-	-	-	-
**Employment status**						
Unemployed	103	83.74	-	-	-	-
Employed	20	16.26	-	-	-	-
**Grant recipient**						
Disability grant	53	43.09	-	-	-	-
Pension grant	33	26.83	-	-	-	-
No grant	37	30.08	-	-	-	-
**Income**						
No monthly income	19	15.45	-	-	-	-
Employed or grant	104	84.55	-	-	-	-
**Number of children**	-	-	-	-	1.00	0.00, 2.00
**HFI access**						
Secure	65	52.85	-	-	-	-
Insecure	58	47.15	-	-	-	-
**QoL total score**	-	-	231.30	48.59	-	-
**Physical**	-	-	-	-	53.57	46.43, 64.29
**Psychological**	-	-	-	-	54.17	45.83, 62.50
**Social relationships**	-	-	62.53	19.31	-	-
**Environment**	-	-	-	-	62.50	50.00, 71.88

s.d., standard deviation; IQR, interquartile range; HFI, household food insecurity; QoL, quality of life.

† , Other refers to relatives, extended family member, children, friends.

A schizophrenia spectrum disorder was the most common diagnosis (52.85%), the median duration of illness being 12 years, with an interquartile range (IQR) of 6.00 to 19.00. Of the 16 (13.01%) participants who had a comorbid illness, 14 (87.50%) had a psychiatric illness, while two (12.50%) had a medical illness only.

Table 2 shows the HFIAS categories, with 47.15% of participants having reported food insecurity of different levels, with most being moderately food insecure (21.95%). The results showed that the overall prevalence of food insecurity in this study was 47.15%, with 13.01%, 21.95% and 12.19% being mildly, moderately and severely food insecure, respectively.

**TABLE 2:** Household Food Insecurity Access Scale categories (*N* = 123).

Variable	*n*	%
**HFIAS access categories**		
Secure	65	52.85
Mildly insecure	16	13.01
Moderately insecure	27	21.95
Severely insecure	15	12.19
**HFIAS access dichotomised**		
Secure	65	52.85
Insecure	58	47.15

HFIAS, Household Food Insecurity Access Scale.

Data for internal consistency regarding the WHOQOL-BREF and HFIAS are presented in Table 3. Internal consistency, at greater than 0.70, was within acceptable limits, except for the social relationships domain of the WHOQOL-BREF.

**TABLE 3:** Internal consistency of data collection instruments.

Tool	Domain	MacDonald’s omega
WHOQOL	Total score	0.91
	Physical	0.79
	Psychological	0.76
	Social Relationships	0.59
	Environment	0.77
HFIAS	-	0.82

WHO QOL-BREF: World Health Organization Quality of Life questionnaire; HFIAS, Household Food Insecurity Access Scale.

Table 4 shows the bivariate analysis, where food insecurity was significantly associated with younger age (*p* = 0.02), having no monthly income (*p* = 0.01), a comorbid psychiatric diagnosis (*p* = 0.02) and a poorer overall QoL (*p* < 0.001). Participants who were food insecure scored lower in total score of QoL (*p* < 0.001), as well as in all the domains of QoL: physical (*p* < 0.035), psychological (*p* < 0.001), social relationships (*p* < 0.001) and environment (*p* < 0.001).

**TABLE 4:** Bivariate analysis comparing participant characteristics by food security.

Variable	Food security												*p*	Test
	Secure (*N* = 65)y						Insecure (*N* = 58)							
	*n*	%	Mean	s.d.	Median	IQR	*n*	%	Mean	s.d.	Median	IQR
**Age (years)**	-	-	53.22	13.42	-	-	-	-	47.43	13.60	-	-	0.02	Two sample *t*-test
**Race**	-	-	-	-	-	-	-	-	-	-	-	-	0.89	Fisher’s exact
Black or African people	17	26.15	-	-	-	-	15	25.86	-	-	-	-	-	-
White people	4	6.15	-	-	-	-	3	5.17	-	-	-	-	-	-
Indian people	33	50.77	-	-	-	-	27	46.55	-	-	-	-	-	-
Coloured people	11	16.92	-	-	-	-	13	22.41	-	-	-	-	-	-
**Marital status**	-	-	-	-	-	-	-	-	-	-	-	-	0.95	Pearson’s Chi-squared
Single	36	55.38	-	-	-	-	31	53.45	-	-	-	-	-	-
Married	17	26.15	-	-	-	-	15	25.86	-	-	-	-	-	-
Divorced	12	18.46	-	-	-	-	12	20.69	-	-	-	-	-	-
**Living with**	-	-	-	-	-	-	-	-	-	-	-	-	0.55	Fisher’s exact
Alone	3	4.62	-	-	-	-	6	10.34	-	-	-	-	-	-
Spouse or child	15	23.08	-	-	-	-	12	20.69	-	-	-	-	-	-
Parents or siblings	31	47.69	-	-	-	-	23	39.66	-	-	-	-	-	-
Other†	16	24.62	-	-	-	-	17	29.31	-	-	-	-	-	-
**Area of residence**	-	-	-	-	-	-	-	-	-	-	-	-	1.00	Fisher’s exact
Urban	61	93.85	-	-	-	-	54	93.10	-	-	-	-	-	-
Rural	4	6.15	-	-	-	-	4	6.90	-	-	-	-	-	-
**Primary diagnosis**	-	-	-	-	-	-	-	-	-	-	-	-	0.71	Fisher’s exact
Schizophrenia spectrum	36	55.38	-	-	-	-	29	50.00	-	-	-	-	-	-
Major Depressive Disorder	15	23.08	-	-	-	-	17	29.31	-	-	-	-	-	-
Bipolar Mood Disorder	8	12.31	-	-	-	-	4	6.90	-	-	-	-	-	-
Anxiety Disorder	1	1.54	-	-	-	-	2	3.45	-	-	-	-	-	-
Other	5	7.69	-	-	-	-	6	10.34	-	-	-	-	-	-
**Duration of illness**	-	-	15.09	11.10	-	-	-	-	14.10	11.70	-	-	0.63	Two sample *t*-test
**Comorbid psych diagnosis**	-	-	-	-	-	-	-	-	-	-	-	-	0.02	Fisher’s exact
No	62	95.38	-	-	-	-	47	81.03	-	-	-	-	-	-
Yes	3	4.62	-	-	-	-	11	18.97	-	-	-	-	-	-
**Comorbid medical diagnosis**	-	-	-	-	-	-	-	-	-	-	-	-	0.03	Fisher’s exact
No	61	93.85	-	-	-	-	46	79.31	-	-	-	-	-	-
Yes	4	6.15	-	-	-	-	12	20.69	-	-	-	-	-	-
**Highest Grade passed**	-	-	-	-	12.00	10.00, 12.00	-	-	-	-	12.00	10.00, 12.00	0.76	Wilcoxon rank-sum
**Tertiary education**	-	-	-	-	-	-	-	-	-	-	-	-	0.06	Pearson’s Chi-squared
No	43	66.15	-	-	-	-	47	81.03	-	-	-	-	-	-
Yes	22	33.85	-	-	-	-	11	18.97	-	-	-	-	-	-
**Employment status**	-	-	-	-	-	-	-	-	-	-	-	-	0.83	Pearson’s Chi-squared
Unemployed	54	83.08	-	-	-	-	49	84.48	-	-	-	-	-	-
Employed	11	16.92	-	-	-	-	9	15.52	-	-	-	-	-	-
**Grant recipient**	-	-	-	-	-	-	-	-	-	-	-	-	0.14	Pearson’s Chi-squared
Disability grant	29	44.62	-	-	-	-	24	41.38	-	-	-	-	-	-
Pension grant	21	32.31	-	-	-	-	12	20.69	-	-	-	-	-	-
No grant	15	23.08	-	-	-	-	22	37.93	-	-	-	-	-	-
**Income**	-	-	-	-	-	-	-	-	-	-	-	-	0.01	Pearson’s Chi-squared
No income	5	7.69	-	-	-	-	14	24.14	-	-	-	-	-	-
Employed or grant	60	92.31	-	-	-	-	44	75.86	-	-	-	-	-	-
Number of children	-	-	-	-	1.00	0.00, 2.00	-	-	-	-	1.00	0.00, 2.00	0.69	Wilcoxon rank-sum
QoL total score	-	-	252.29	41.48	-	-	-	-	207.76	45.35	-	-	< 0.001	Two sample *t*-test
Physical	-	-	-	-	57.14	50.00, 64.29	-	-	-	-	53.57	46.43, 60.71	0.035	Wilcoxon rank-sum
Psychological	-	-	-	-	62.50	50.00, 66.67	-	-	-	-	50.00	41.67, 54.17	< 0.001	Wilcoxon rank-sum
Social relationships	-	-	69.23	16.13	-	-	-	-	55.03	19.93	-	-	< 0.001	Two sample *t*-test
Environment	-	-	-	-	68.75	59.38, 75.00	-	-	-	-	53.12	40.62, 62.50	< 0.001	Wilcoxon rank-sum

s.d., standard deviation; QoL, quality of life; IQR, interquartile range.

† , Other refers to relatives, extended family member, children, friends.

The effect size for significantly associated variables is presented in Table 5.

**TABLE 5:** Effect size.

Variable	Cohen’s *d*	95% CI
Age (years)	0.43	0.07, 0.79
Comorbid Psychiatric Diagnosis	−0.46	−0.82, −0.10
Comorbid Medical Diagnosis	−0.44	−0.80, −0.08
Income	0.46	0.10, 0.82
QoL total score	1.03	0.65, 1.40
Physical	0.40	0.04, 0.76
Psychological	0.62	0.26, 0.98
Social Relationships	0.79	0.42, 1.15
Environment	1.33	0.93, 1.72

s.d., standard deviation; QoL, quality of life; CI, confidence interval.

## Discussion

The rationale for conducting this study in women with mental illness was because of the global evidence that shows that women and people with mental illnesses are more vulnerable to food insecurity.^24,28^ While having adequate access to food is a basic human right in SA, this study found that 47% of women with mental illnesses who were attending a psychiatric unit in KZN reported being food insecure. Food insecurity was significantly associated with a younger age, no source of monthly income, a comorbid psychiatric illness, and a poorer QoL in all domains of the (WHOQOL-BREF) scale.

There is a wide variation in the published prevalence of food insecurity in people with mental illnesses, ranging from 9.9% to 85.7%.^55^ The overall prevalence of food insecurity in this study is similar to that reported in two recent international meta-analyses that found a prevalence of 40%^55^ and 41%^34^ in people with severe mental illnesses. These meta-analytic prevalence estimates are not directly comparable to the prevalence in our study because of the different measuring tools, and as they did not stratify their results by gender. The tools used to investigate food insecurity also differed, with the Teasdale et al. study using the USDA Household Food Insecurity Survey.^55^ In Smith et al., a number of tools were used, including the US Adult Food Security Survey Module, the HFIAS, a two item screen for families at risk of food insecurity and various other tools.^34^ The differences in tools used also make it difficult to make definitive comparisons between the data.

The findings of this study confirm the global reports of an increased prevalence of food insecurity in mentally ill people compared to those without from the corresponding population. The prevalence of food insecurity in this study is almost double that reported among the general population of SA, which ranges between 26% and 29%.^10,11^ The prevalence in this study may be an overestimation of food insecurity between those with and without mental illnesses in SA given that the previous rates^10,11^ did not stratify by gender. However, it may also be a true reflection of the high prevalence of food insecurity in women with mental illnesses, as females in female headed households in SA were reported to earn less, and were more household insecure than those headed by males.^31,32^ An analysis of a national general household survey found that black female-headed households were the most food insecure households.^33^

No association was found between household size and food insecurity, with some studies indicating that households with a higher number of family members were found to have higher odds of food insecurity than those with fewer members.^56,57^ A possible explanation is that 55% of the women in this study were single, and 44% were living with their parents or siblings.

In this study, almost 70% of the participants were recipients of social or disability grants; SA having one of the most extensive systems of publicly funded social assistance programmes in the world in the form of cash transfers for those with chronic illness and advanced age.^58,59^ Social grants remain one of the ways government provides relief in an attempt to decrease poverty.^60^ A previous study found that social grants have significantly reduced poverty levels in areas with high poverty levels and female-headed households.^61^ However, Chakone et al. suggested that with increasing food prices, social grants alone may not be enough to alleviate or improve food insecurity in SA,^23^ this being confirmed by our results.

We did not find an association between race, ethnicity and food insecurity, this aspect having been indicated in previous studies.^22,62,63^ This lack of association in our study is possibly because of the historical legacy of the racial residential delineations by ethnic groups that largely persist today in the hospitals catchment area, and the number of participants being too small to detect differences between the ethnic groups.

Our finding of a significant association between comorbid psychiatric disorders and food insecurity is in keeping with previous studies, which found that severe mental disorders are generally chronic in nature, chronic conditions often being associated with higher odds of food insecurity.^64^ A study conducted in Canada also found that the odds of experiencing food insecurity increased with an increase in the number of chronic conditions.^65^

The association between severe mental illnesses and QoL is well established in the literature, with findings suggesting that affected individuals have a poorer QoL than those who are not.^46^ In our study, women who were food insecure scored poorly in all the domains of quality of life, with similar findings being reported by other researchers in the US and Australia.^21,66^ Authors have suggested that a possible explanation may be that food insecurity affects other aspects of an individual’s life, such as the fulfilment of social or societal roles that are important for attaining a better QoL.^21^

While this study makes an important contribution to the literature on food security on women with mental illnesses in SA, several limitations are acknowledged. The study only being conducted in one hospital and the small sample size may have limited the statistical power and contributed to some of the negative findings. It would have been helpful to collect data on total household income to investigate the association between total monthly household income and food insecurity. Use of a male comparative group would have assisted in making conclusive gender comparisons. The internal consistency of the social relationships domain was low; therefore, the associations found should be interpreted with caution. Another study conducted on an all-female population also reported low internal consistency in this domain.^67^ This suggests that personal relationships and friendships – both assessed within this domain – might represent distinct types of relationships for women and may need to be evaluated separately.

## Conclusion

Our study sought to describe the prevalence of food insecurity and its association with socio-demographic factors, clinical factors and QoL in women with mental illnesses attending a psychiatric hospital in KZN. We found a high prevalence of food insecurity among our participants which highlights the need to screen women with mental illnesses for food insecurity, as it may have a significant impact on health (including mental health), nutrition, behaviour and QoL. Longitudinal studies are needed to determine food insecurity in women with mental illnesses on a broader scale to establish the extent of the problem and therefore inform interventions that can be directed at this vulnerable population group. Qualitative research looking into the lived experiences of women experiencing food insecurity is also needed.
